# Does early surgery improve outcomes for periprosthetic fractures of the hip and knee? A systematic review and meta-analysis

**DOI:** 10.1007/s00402-020-03739-2

**Published:** 2021-02-08

**Authors:** L. Farrow, A. D. Ablett, H. W. Sargeant, T. O. Smith, A. T. Johnston

**Affiliations:** 1 Aberdeen Royal Infirmary, Foresterhill, Aberdeen, AB25 2ZG UK; 2 Institute of Medical Sciences, University of Aberdeen, Foresterhill, Aberdeen, AB25 2ZD UK; 3 Nuffield Department of Rheumatology, Orthopaedics and Musculoskeletal Sciences, Botnar Research Centre, University of Oxford, Windmill Road, Oxford, OX3 7LD UK

**Keywords:** Trauma, Orthopaedics, Periprosthetic, Fracture, Hip, Knee, Arthroplasty, Delay, Time to surgery, Systematic review

## Abstract

**Introduction:**

Previous evidence has established that early surgery is beneficial to improve outcomes for individuals with native hip fractures in the elderly population. Patients who sustain a periprosthetic fracture have been demonstrated to have similar demographics and outcomes as those with native fractures around the hip and knee. We therefore set out to determine if there is a similar difference in perioperative outcomes between early and delayed surgery for periprosthetic fractures of the hip and knee through a systematic review and meta-analysis.

**Methods:**

Literature search outputs were screened for studies meeting the inclusion criteria. The groups of early surgery and delayed surgery were defined by study authors. The primary outcome measure was 30 day mortality. Where there was sufficient study homogeneity, a random-effects meta-analysis was performed. Individual study risk of bias was assessed using the ROBINS-I criteria, with the GRADE criteria used for independent outcome evaluation. The review protocol was registered on PROSPERO prior to commencement (Registration number CRD42019149360).

**Results:**

The inclusion criteria was met in 11 studies (*n* = 3006). Mean time to surgery from admission for reporting studies was 64 h. 59.6% patients underwent early surgery as defined by the study authors. We identified a significantly lower risk of 30 day mortality for those with early surgery versus delayed surgery (RR 0.21; 95% CI 0.05, 0.90; *p* = 0.04, *n* = 2022). There were also significantly better outcomes for early versus delayed surgery regarding: medical complications, length of stay, transfusion risk, and reoperation. The quality of evidence for all the individual outcomes was low or very low.

**Conclusions:**

There is evidence that delaying surgery in those with periprosthetic fractures of the hip and knee has a deleterious impact on mortality and other important patient outcomes. There are, however, notable limitations to the existing available literature, with further appropriately designed large-scale studies required to confirm these findings.

**Supplementary Information:**

The online version contains supplementary material available at 10.1007/s00402-020-03739-2.

## Introduction

Periprosthetic fractures around the hip and knee are an emerging problem across the orthopaedic community. Rates are expected to rise significantly in the future associated with dramatic increases in the amount of primary and revision arthroplasties performed, as well as an ageing population [22].

The patient cohort is similar to those who sustain a fragility fracture of the hip or distal femur [4, 20], where there has been previous interest in time to surgery. There is now strong evidence for increased patient complications, including a higher mortality rate, when surgery is delayed in this setting [20, 25, 27, 28]. Many hip fracture guidelines now incorporate recommendations regarding minimal delay to surgery, typically within 36–48 h of admission [2, 23]. Current UK national guidance suggests that all frail patients requiring surgery should have this performed within 36 h, which includes those with fragility fractures of the distal femur [5].

Given the similar reported mortality and morbidity for periprosthetic hip and knee fractures compared to native fractures [4, 20], it is perhaps surprising that periprosthetic fractures appear to often be associated with a significant delay from injury to theatre [12, 14, 16]. This is possibly related to difficulties regarding service provision for such fractures, where particular surgical expertise and specific implants are required. These challenges could, however, be overcome with appropriate planning and recognition if a clinical demand for prompt surgery was recognised. This has previously been seen in hip fracture surgery where national registries have demonstrated significant reductions in the time to theatre for the majority of patients [23, 29].

This systematic review and meta-analysis aimed to determine if delays to surgery for patients suffering periprosthetic fractures around hip or knee implants were associated with complication rates.

## Materials and methods

The study was reported in accordance with the PRISMA (Preferred Reporting Items for Systematic Reviews and Meta-Analyses) guidance [19]. The review protocol was registered on the international prospective register of systematic reviews (PROSPERO) prior to commencement (Registration number CRD42019149360). Hip and knee periprosthetic fractures were included together due to the relative scarcity of cases, with both conditions occurring in the same patient cohort, and having similar reported outcomes for native hip and distal femoral fractures [20, 25].

### Search strategy

Medline, EMBASE, and the Cochrane Central Registry of Controlled Trials were investigated for relevant outputs using the OvidSP search platform. Additional searches using the Google search engine, the WHO clinical trial registry, clinicaltrials.gov, and the OpenGrey database were also performed to ensure that all potentially eligible materials were identified; reference lists of relevant articles were also screened. All electronic searches were undertaken from database inception to October 2019. The only limit placed on the search strategy was articles in the English language. The full electronic search strategy is presented in “Appendix 1”.

### Eligibility criteria

Included studies were non-review research articles (clinical trials, case series, and other observational study methods) reporting on clinical outcomes relating to periprosthetic fractures around the hip and knee. This had to include analysis regarding time to surgery. The intervention and control groups were early surgery and delayed surgery (as defined by the study authors e.g., < 24 h and ≥ 24 h) respectively. Only studies that considered time to surgery as an independent categorical variable were included. Studies including periprosthetic fractures in areas other than the hip and knee, periprosthetic patella fractures, non-operative management, open fractures, and neurovascular injury were excluded. No restrictions were placed on the level of evidence presented through individual studies or the study design.

### Study identification

Studies were identified from screened abstracts of potentially appropriate studies which met the eligibility criteria by two independent reviewers (LF and AA). Selected studies then proceeded to full-text assessment where a final decision on suitability was made. Any discrepancy regarding study eligibility was decided by reviewer discussion.

### Data extraction

Two assessors (AA and HS) independently extracted relevant data from each study using standardised data forms for both patient demographics and outcomes. Demographic data included: study year, study design, number participants, gender, age, American Society of Anaesthesiologists physical status classification (ASA), Charlson Co-morbidity Index, osteoporosis, dementia, time from initial surgery to fracture, type of periprosthetic fracture (hip/knee/both), Vancouver/Unified classification system, time to surgery, type of surgery (revision/osteosynthesis/both), anaesthetic type, duration of surgery, and intervention/control group (if comparing time to surgery). In the instance of missing data, attempts were made to contact study authors. Outcome data fields are detailed below.

### Outcome measures

The primary outcome measure was 30 day mortality. Secondary outcome measures included: 1-year mortality, length of stay, transfusion, all-cause medical complications (respiratory, e.g., pneumonia, cardiovascular, e.g., myocardial infarction/stroke, renal, e.g., urinary tract infection and acute kidney injury, and sepsis), surgical site infection, and all-cause reoperation (infection, dislocation, and implant failure/fracture).

### Quality assessment

Risk of bias in observational studies was assessed using The Risk of Bias in Non-randomised Studies-of Interventions (ROBINS-I) tool [26]. This was performed independently by two reviewers (LF and AA). Bias assessment was also performed for each individual outcome using the Grading Quality of Evidence and Strength of Recommendations (GRADE) criteria [3].

### Statistical analysis

Where there were a minimum of two studies categorically assessing time to surgery as an independent variable, with sufficient homogeneity regarding the study design, intervention, and population, a meta-analysis was undertaken using Revman 5.3 [1]. For cases where the included studies did not meet the criteria for meta-analysis, a narrative review was performed. Where meta-analysis was performed, a random-effects model was utilised. Standard mean difference (SMD) was calculated for continuous outcomes, with relative risk (RR) for dichotomous variables. In all analyses *p* < 0.05 denoted statistical significance. Analyses are presented with 95% confidence intervals.

## Results

**Fig. 1:**
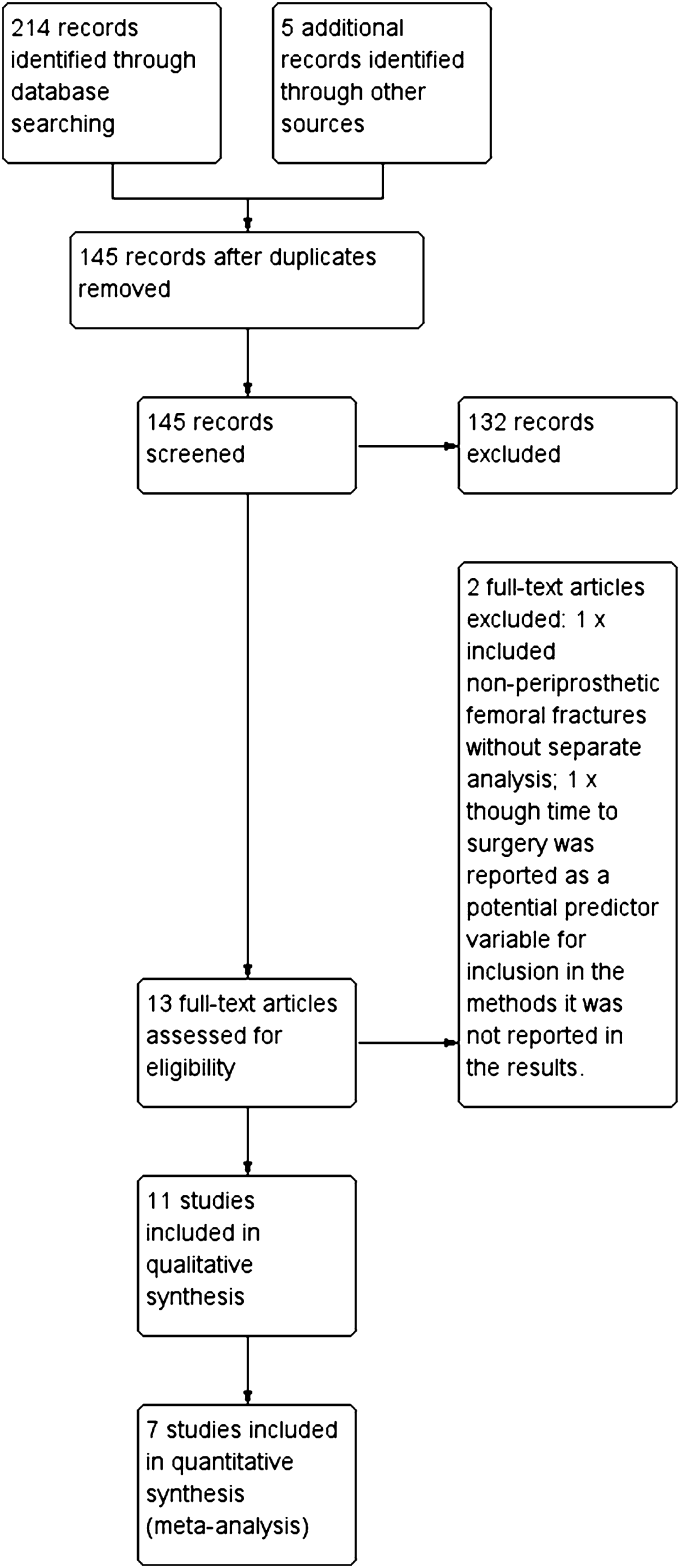
Flow diagram of study selection process

The search results are summarised in Fig. 1. A total of 144 studies were identified, with 13 undergoing full-text review. 11 articles [4, 6–8, 10, 12, 13, 15, 16, 18, 24] were determined to be eligible for study inclusion.

### Study characteristics

A summary of included studies is shown in Table 1. A total of 3006 participants were included. Mean time to fracture from index procedure was 7.6 years for reporting studies. Mean time to surgery from admission for reporting studies was 64 h. In the two studies that reported dichotomous time to surgery at 24 h [6, 7], 46.9% (402/857) and 77.9% (377/484) participants were found to have received surgery within 24 h respectively. Three studies reported dichotomous time to surgery at 48 h [8, 18, 24], with 31.1% (56/180), 60.1% (409/681), and 216/263 (82%) of individuals receiving surgery within this time frame. All 11 articles included in the study were of a retrospective cohort design. Individual risk of bias assessment was performed for each study according to the ROBINS-I tool, with results shown in supplementary Table 1. Results for the GRADE analysis are reported for individual outcomes, with a summary contained within supplementary table 2.

**Table 1:** Characteristics of included studies

Paper	Study	*N*	Sex (F)	Mean age (years) [SD]	ASA	Time from initial surgery to fracture (years)	Type of periprosthetic fracture (hip/knee/both)	Time to surgery from admission	Type of surgery (revision/osteosynthesis/both)	Duration of surgery	Intervention/control (time to surgery from admission if dichotomised comparison)
Bhattacharyya et al., 2007	RCC	106	70 (66%)	79.1 [10.7]		Mean 7.6	Both	Mean (SD) 2.3 (1.6) days	Both	Not reported	I: surgery ≤ 48 hours C: surgery > 48 hours
Boddapati et al., 2019 (1)	RC	857	503 (58.7%)	< 60 = 22.4%, 60–70 = 20.8%, 71–80 = 26.8% > 80 = 30.0%	1 or 2 = 32.6%	Not reported	Hip	I: (≤ 24 hours) = 46.9%) C: (> 24 hours ) = 53.1%	Revision	≤ 120 min = 28.4%	I: surgery ≤ 24 hours C: surgery > 24 hours
Boddapati et al., 2019 (2)	RC	484	329 (68%)	< 60 = 22.7%, 60–70 = 32.9%, 71–80 = 29.8%, > 80 = 14.7%	1 or 2 = 38.8%	Not reported	Knee	I: (≤ 24 hours) = 77.9%) C: (> 24 hours) = 22%	Revision	≤ 120 min = 39.0%	I: SURGERY ≤ 24 hours C: Surgery > 24 hours
Bovonratwet et al., 2019	RC	681	437 (64.2%)	I: 73.5 [11.8] C: 75 [13.1]	Mean (SD) I: 2.8 (0.6); C: 3 (0.6)	Not reported	Hip	I: (< 48 hours) = 60.1% C: (≥ 48 hours) = 39.9%	Both	Mean (SD) I: (< 48 hours) = 167.5 (75.9) C: (≥ 48 hours) = 177.6 (74.6)	I: surgery < 48 hours C: surgery ≥ 48 hours
Fuchtmeier et al., 2015	RC	121	85 (70.2%)	75.5 [11.6]	1 or 2 = 33.9%	Mean 6.3	Hip	Mean 32 h	Both	Mean 142 min	Not applicable
Griffiths et al., 2013	RC	60	39 (65%)	78 [range 51–98]	Median = 3	Mean 8.7	Hip	Mean 4 days	Both	Not reported	I: surgery ≤ 72 hours C: surgery > 72 hours (subgroup)
Hoellwarth et al., 2018	RC	140	Not reported	80 [9.1]	Not reported	Not reported	Knee	Mean (SD): 1.6 (1.5) days	Both	Not reported	I: surgery ≤ 48 hours C: surgery ≥ 72 hours (subgroup)
Jennison et al., 2018	RC	32	16 (50%)	76.5 [range 57–96]	1 or 2 = 37.5%	Not reported	Hip	Mean 105 h	Both	Not reported	Not applicable
Johnson-Lynn et al., 2015	RC	82	42 (51.2%)	78.3 [range 46–93]	Mean = 2.8	Mean 8.8	Hip	Mean 4.2 days	Both	Mean 3.3 h	Not applicable
Ro Lee et al., 2018	RC	263	210 (79.8%)	73.9 [12]	Not reported	Not reported	Knee	Mean (SD) 1.6 (2.1) days	Both	Mean (SD) = 133.1 (52) mins	I: surgery ≤ 48 hours C: surgery > 48 hours
Sellan et al., 2017	RC	180	130 (72.2%)	79.2 [10.1]	Mean (SD) I: 3.3 (0.6); C: 3.4 (0.6)	Not reported	Both	I: (≤ 48 hours) = 31.1% C: (> 48 hours) = 68.9%	Both	Mean (SD) = 147 (47) mins	I: surgery ≤ 48 hours C: surgery > 48 hours

*RC* retrospective cohort, *RCC* retrospective case–control, *N* number of participants

### Primary outcome: 30 day mortality

**Fig. 2:**
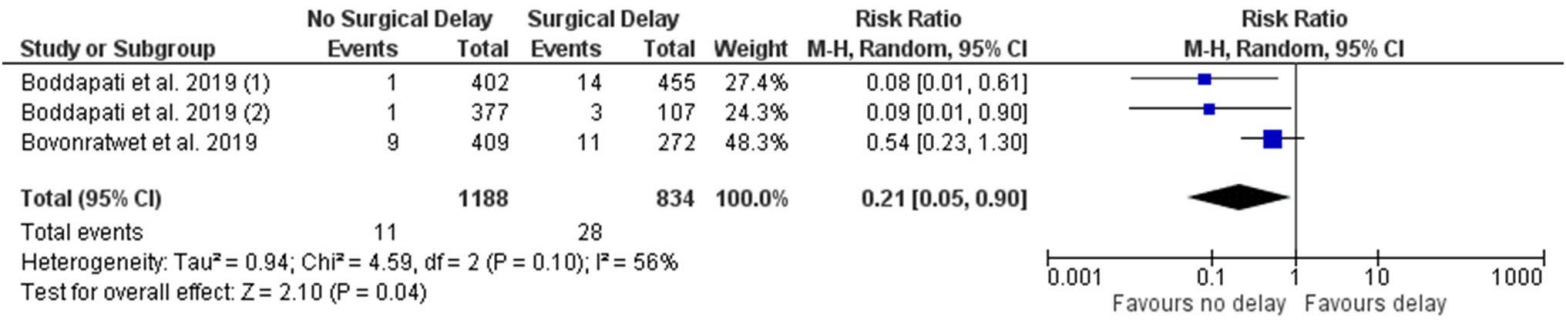
Forest plot of delayed versus early surgery for the outcome of 30 day mortality

Three studies reported 30 day mortality with a dichotomised delay to surgery [6–8]. On meta-analysis, there was a significantly lower risk of 30 day mortality for those with early surgery versus delayed surgery (RR 0.21; 95% CI 0.05, 0.90; *p* = 0.04, *n* = 2022—Fig. 2). All other studies did not report on 30 day mortality. On GRADE assessment, the quality of evidence was found to be very low due to observational design, serious imprecision, and serious inconsistency.

### 1-Year mortality

Three studies reported 1-year mortality with a dichotomised delay to surgery [4, 13, 24]. On meta-analysis, there was a trend towards a lower mortality at 1 year for early versus delayed surgery, but no statistically significant difference (RR 0.61; 95% CI 0.36, 1.03; *p* = 0.06, *n* = 407—supplementary Fig. 1). In addition Fuchtmeier et al. 2015 reported on 1 year mortality and found no significant difference in mean delay to surgery for those with no mortality (21.1 h) and those with mortality (27.2 h); *p* = 0.60. Jennison and Yarlagadda [15] also found no significant difference in 1 year mortality in mean delay to surgery for those with no mortality (109 h) versus those with mortality (104 h); *p* = 0.88. On GRADE assessment, the level of evidence was found to be very low due to observational design, a serious risk of bias, serious imprecision, and serious inconsistency.

### Length of stay

Two studies reported length of stay with a dichotomised delay to surgery as greater than or less than 24 h [6, 7]. On meta-analysis, there was a significantly shorter length of stay for those operated on within 24 h, versus those who waited longer (SMD − 1.03 days; 95% CI − 1.88, − 0.19; *p* = 0.02, *n* = 1341—supplementary Fig. 2). Conversely, Johnson-Lynn et al. [16] found that there was no significant positive correlation between delay to surgery and overall length of stay (Pearson correlation coefficient − 0.1191, *n* = 82). On GRADE assessment, the level of evidence was found to be very low due to observational design, serious imprecision, and serious inconsistency.

### Transfusion

Four studies reported on transfusion rate with a dichotomised delay to surgery [4, 6, 7, 18]. On meta-analysis, there was a significantly lower transfusion rate for those with an early operation, compared to those with a delay to surgery (RR 0.51; 95% CI 0.31, 0.82; *p* =  < 0.001, *n* = 2285—Supplementary Fig. 3). On GRADE assessment, the level of evidence was found to be very low due to observational design and serious inconsistency.

### Medical complications (all cause)

Five studies reported on medical complications with a dichotomised delay to surgery [6–8, 18, 24]. On meta-analysis, there was a significantly lower medical complication rate for those with early surgery versus delayed surgery (RR 0.58; 95% CI 0.42, 0.82; *p* = 0.002, *n* = 2465—Supplementary Fig. 4). Griffiths et al. 2013 [12] also reported on the influence of surgical delay on post-operative complications (although this included both medical and surgical complications). They found that patients with no complications had a significantly shorter surgical delay (2.82 days) compared to those with complications (5.41 days) (*p* = 0.02). Johnson-Lynn et al. [16], on the other hand, did not find a correlation between the number of post-operative complications and an increasing delay to surgery (Pearson correlation coefficient − 0.0444, *n* = 82). On GRADE assessment, the level of evidence was found to be very low due to observational design and serious inconsistency.

### Surgical site infection

Five studies reported on surgical site infection with time to surgery as an independent categorical variable [6–8, 18, 24]. On meta-analysis, there was a trend towards a lower surgical site infection rate with a shorter delay to surgery, but this was not statistically significant (RR 0.61; 95% CI 0.36, 1.03; *p* = 0.06, *n* = 2465—Supplementary Fig. 5). On GRADE assessment, the level of evidence was found to be low due to observational design.

### Reoperation (all cause)

Three studies reported on all-cause reoperation with a dichotomised delay to surgery [6–8]. On meta-analysis, there was a significantly lower reoperation rate for those with early surgery compared to delayed surgery (RR 0.63, 95% CI 0.45, 0.89; *p* =  < 0.001, *n* = 2022—Supplementary Fig. 6). There were insufficient data available to split reoperation by cause (e.g., infection, dislocation, implant failure, or fracture). On GRADE assessment, the level of evidence was found to be low due to observational design.

## Discussion

This systematic review and meta-analysis found that there is weak evidence, as defined by the GRADE criteria, to suggest a delay to surgery for patients with a periprosthetic fracture of either the hip or knee is associated with worse patient outcomes. This included increased 30 day mortality, greater likelihood of medical complications, longer length of stay, greater risk of transfusion, and reoperation. There were also trends identified towards a greater risk of 1 year mortality and surgical site infection.

These findings are similar to those previously identified in native hip [25] and distal femoral [20] fractures, where prompt surgery has been associated with improved outcomes. As demonstrated by the low-quality evidence identified during this study, the links in the periprosthetic fracture setting are less concrete, with a clear need for further research to confirm our findings. Given the study design limitations of the current evidence, it is difficult to discern whether the associations observed in this work are due to causal effect from delay to surgery, or patient selection bias. Nevertheless, taking into account the similarities in the hip fracture and periprosthetic fracture cohorts [4], clinical reasoning would support the hypothesis that the impact of a delay to surgery would be similar for both groups. Current UK national guidance suggests that all frail patients should be managed with urgent surgery to quickly restore mobility and reduce associated complications [5].

One of the potential issues with managing time to surgery for periprosthetic fractures is that there are currently significant service provision limitations that would have to be addressed prior to implementing improvements in surgical delay. This is particularly true for revision surgery, where specialist hip surgeon knowledge and skills are often required, and may not be immediately available. A number of studies included in this analysis confirmed that patients undergoing revision surgery had longer waiting times compared to those treated with fixation alone [8, 13, 15]. Given that revision surgery for Vancouver B2 fractures has already been associated with mortality and morbidity benefits compared to fixation alone [17], these results may actually be even more pronounced when taking into account the impact of surgical delay. Examination of different models of service provision, such as a specific arthroplasty on-call service providing reliable access to surgical expertise, would undoubtedly be beneficial in the ultimate implementation of expedited surgery for these patients.

When considering future studies within this area, one of the issues demonstrated throughout our analysis is a lack of consistent reporting and outcomes between studies. Despite 11 articles included in the systematic review, only 7 were suitable for inclusion within a meta-analysis. In addition, many of these studies utilised different methodologies and reported on different outcomes. Development of a consensus core dataset such as that suggested by Khan et al. [17] in their systematic review would be of benefit to ensure consistent reporting across studies, and aid in conducting meaningful comparisons.

Establishment of national or international periprosthetic fracture registries would also be of benefit in further understanding the impact of time to surgery for these injuries given their relatively low incidence. Such registries would allow for the completion of large-scale cohort studies, which would be particularly useful given the difficulties in patient recruitment and ethical concerns for performing randomised-controlled trials in this setting. Techniques such as propensity matching for known confounders would help mitigate the potential bias from patient selection; particularly as a number of other factors potentially influential in periprosthetic fracture outcomes have been previously described [11, 17, 21]. The use of large-scale registry data should also allow for the separation of periprosthetic knee and periprosthetic hip fracture patients into individual cohorts, as a previous report has suggested the possibility of difference in demographics and outcome between the two cohorts [9]. Using the results from our analysis, we have calculated an estimated sample size for such a study examining dichotomised time to surgery and 30 day mortality would require 1170 participants to appropriately investigate this primary outcome (https://www.clincalc.com/stats/samplesize.aspx). None of the current included studies met this requirement (range 32–857 participants).

## Conclusion

Current evidence suggests that delaying hip and knee periprosthetic fracture surgery is associated with higher patient morbidity and mortality. Given the low quality of this evidence, further large-scale studies, with appropriate adjustment for confounding bias, are required to confirm these findings. Understanding the service provision barriers that prevent early surgery for these patients would help to design interventions to reduce delay and consequently improve key patient healthcare outcomes.

## Supplementary Information

Below is the link to the electronic supplementary material.

Supplementary file1 (DOC 24 KB)

Supplementary file2 (DOCX 12 KB)

Supplementary figure 1 – Forest plot of delayed versus early surgery for the outcome of 1 year mortality (PNG 10 KB)

Supplementary figure 2 – Forest plot of delayed versus early surgery for the outcome of length of stay (PNG 9 KB)

Supplementary figure 3 - Forest plot of delayed versus early surgery for the outcome of pin transfusion (PNG 10 KB)

Supplementary figure 4 - Forest plot of delayed versus early surgery for the outcome of medical complications (all cause) (PNG 10 KB)

Supplementary figure 5 – Forest plot of delayed versus early surgery for the outcome of surgical site infection (PNG 10 KB)

Supplementary figure 6 - Forest plot of delayed versus early surgery for the outcome of reoperation (all cause) (PNG 10 KB)

Supplementary file9 (DOCX 17 KB)

Supplementary file10 (DOCX 14 KB)

Supplementary file11 (DOC 63 KB)
