# Supplementary material for: Does early surgery improve outcomes for periprosthetic fractures of the hip and knee? A systematic review and meta-analysis
Source: Arch Orthop Trauma Surg. 2021 Feb 8;141(8):1393–400. doi: 10.1007/s00402-020-03739-2 (PMC8295128; doi:10.1007/s00402-020-03739-2)
Supplement: Supplementary file 1 — Supplementary file1 (DOC 24 KB) [file 402_2020_3739_MOESM1_ESM.doc]

**Appendix 1.**

**Search strategy**

Medline 1946-Present & EmBase 1947-Present & Cochrane registry of controlled trials via the Ovid-SP search engine. Only other limits were English language only.

1. Periprosthetic
2. Fracture*
3. 1 and 2
4. Time to surgery
5. delay
6. 4 or 5
7. Hip
8. Knee
9. 7 or 8
10. 3 and 6 and 9
11. Remove duplicates from 10
