# Supplementary material for: Does early surgery improve outcomes for periprosthetic fractures of the hip and knee? A systematic review and meta-analysis
Source: Arch Orthop Trauma Surg. 2021 Feb 8;141(8):1393–400. doi: 10.1007/s00402-020-03739-2 (PMC8295128; doi:10.1007/s00402-020-03739-2)
Supplement: Supplementary file 2 — Supplementary file2 (DOCX 12 KB) [file 402_2020_3739_MOESM2_ESM.docx]

**Appendix 2 – results for different types of medical complication**

Medical complications (respiratory)

3 studies (Boddapatti 1&2, Sellan) reported on respiratory complications with time to surgery as an independent categorical variable. On meta-analysis there was a significantly lower respiratory complication rate for those with early surgery versus delayed surgery (RR 0.27; 95% CI 0.10, 0.72; P = 0.01, n = 1521).

Medical complications (cardiovascular)

3 studies (Boddapatti 1&2, Sellan) reported on cardiovascular complications with time to surgery as an independent categorical variable. On meta-analysis there was no significant difference regarding the cardiovascular complication rate for those with early surgery versus delayed surgery (RR 0.60; 95% CI 0.60, 1.54; P = 0.29, n = 1521).

Medical complications (renal)

3 studies (Boddapatti 1&2, Sellan) reported on renal complications with time to surgery as an independent categorical variable. On meta-analysis there was a trend towards a lower risk of renal complications for those without surgical delay, but this was not statistically significant (RR 0.34; 95% CI 0.12, 1.00; P = 0.05, n = 1521).

Medical complications (sepsis)

2 studies (Boddapatti 1&2) reported on sepsis rate with a dichotomised delay to surgery as greater than or less than 24 hours. On meta-analysis there was a significantly lower risk of sepsis for patients with no surgical delay versus those with a surgical delay (RR 0.09; 95% CI 0.02, 0.47; P = 0.005, n = 1341).
