# Supplementary figures and images for: Does early surgery improve outcomes for periprosthetic fractures of the hip and knee? A systematic review and meta-analysis
Source: Arch Orthop Trauma Surg. 2021 Feb 8;141(8):1393–400. doi: 10.1007/s00402-020-03739-2 (PMC8295128; doi:10.1007/s00402-020-03739-2)

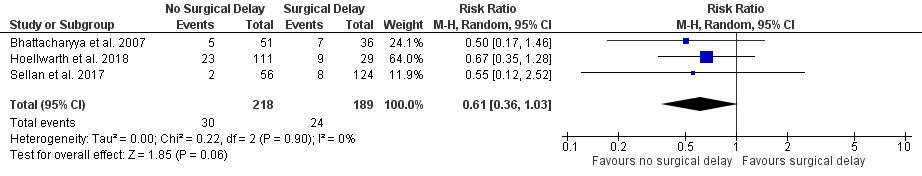

Supplement: Supplementary file 3 — Supplementary figure 1 – Forest plot of delayed versus early surgery for the outcome of 1 year mortality (PNG 10 KB) [file 402_2020_3739_MOESM3_ESM.png]

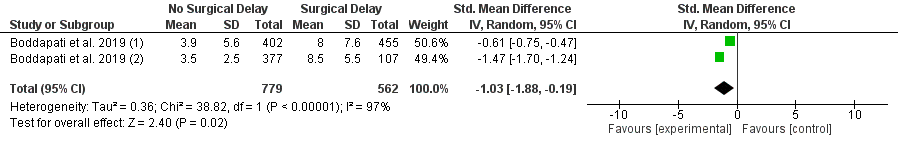

Supplement: Supplementary file 4 — Supplementary figure 2 – Forest plot of delayed versus early surgery for the outcome of length of stay (PNG 9 KB) [file 402_2020_3739_MOESM4_ESM.png]

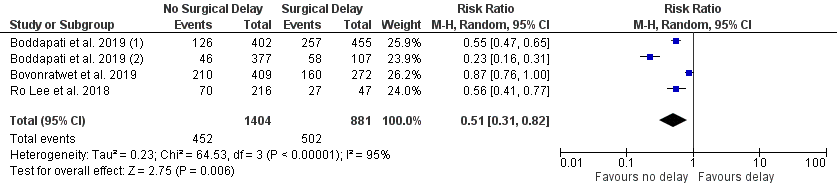

Supplement: Supplementary file 5 — Supplementary figure 3 - Forest plot of delayed versus early surgery for the outcome of pin transfusion (PNG 10 KB) [file 402_2020_3739_MOESM5_ESM.png]

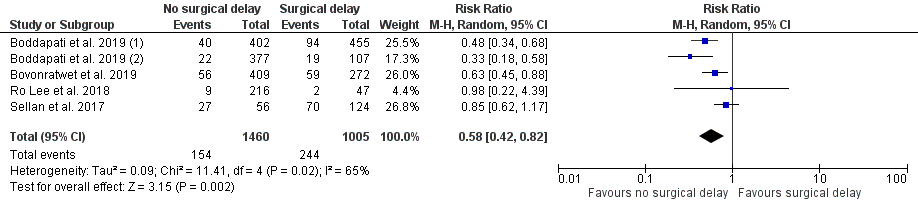

Supplement: Supplementary file 6 — Supplementary figure 4 - Forest plot of delayed versus early surgery for the outcome of medical complications (all cause) (PNG 10 KB) [file 402_2020_3739_MOESM6_ESM.png]

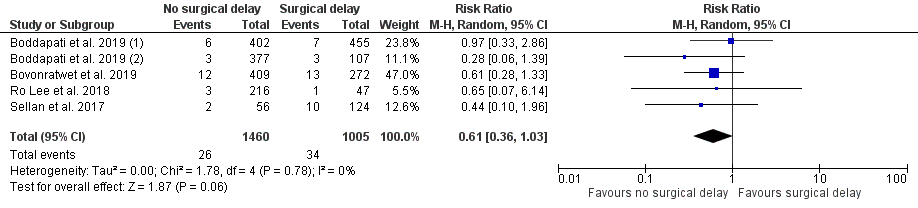

Supplement: Supplementary file 7 — Supplementary figure 5 – Forest plot of delayed versus early surgery for the outcome of surgical site infection (PNG 10 KB) [file 402_2020_3739_MOESM7_ESM.png]

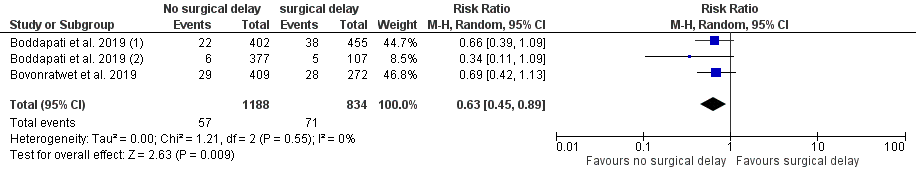

Supplement: Supplementary file 8 — Supplementary figure 6 - Forest plot of delayed versus early surgery for the outcome of reoperation (all cause) (PNG 10 KB) [file 402_2020_3739_MOESM8_ESM.png]
