# Supplementary material for: Does early surgery improve outcomes for periprosthetic fractures of the hip and knee? A systematic review and meta-analysis
Source: Arch Orthop Trauma Surg. 2021 Feb 8;141(8):1393–400. doi: 10.1007/s00402-020-03739-2 (PMC8295128; doi:10.1007/s00402-020-03739-2)
Supplement: Supplementary file 9 — Supplementary file9 (DOCX 17 KB) [file 402_2020_3739_MOESM9_ESM.docx]

**Supplementary table 1.** Risk of bias assessment for individual studies

| ***Observational studies*** | **Bias due to confounding** | **Bias in**  **participant selection** | **Bias in**  **classification**  **of**  **interventions** | **Bias due to**  **departures**  **from intended**  **interventions** | **Bias due to**  **missing data** | **Bias in**  **measurement**  **of outcomes** | **Bias in**  **selection of**  **the reported**  **result** | | **Overall bias** |
| --- | --- | --- | --- | --- | --- | --- | --- | --- | --- |
| Bhattacharyya et al., 2007 | Serious risk | Serious risk | Serious risk | Low risk | Not interpretable | Not interpretable | Not interpretable | Serious risk | |
| Boddapati et al., 2019 (1) | Serious risk | Serious risk | Serious risk | Low risk | Serious risk | Not interpretable | Not interpretable | Serious risk | |
| Boddapati et al., 2019 (2) | Serious risk | Serious risk | Serious risk | Low risk | Serious risk | Not interpretable | Not interpretable | Serious risk | |
| Bovonratwet et al., 2019 | Serious risk | Serious risk | Serious risk | Low risk | Not interpretable | Not interpretable | Not interpretable | Serious risk | |
| Fuchtmeier et al., 2015 | Serious risk | Serious risk | Serious risk | Low risk | Not interpretable | Not interpretable | Not interpretable | Serious risk | |
| Griffiths et al., 2013 | Serious risk | Serious risk | Serious risk | Low risk | Not interpretable | Not interpretable | Not interpretable | Serious risk | |
| Hoellwarth et al., 2018 | Serious risk | Serious risk | Serious risk | Low risk | Not interpretable | Not interpretable | Not interpretable | Serious risk | |
| Jennison et al., 2018 | Serious risk | Serious risk | Serious risk | Low risk | Not interpretable | Not interpretable | Not interpretable | Serious risk | |
| Johnson-Lynn et al., 2015 | Serious risk | Serious risk | Serious risk | Low risk | Not interpretable | Not interpretable | Not interpretable | Serious risk | |
| Ro Lee et al., 2018 | Serious risk | Serious risk | Serious risk | Low risk | Not interpretable | Not interpretable | Not interpretable | Serious risk | |
| Sellan et al., 2017 | Serious risk | Serious risk | Serious risk | Low risk | Not interpretable | Not interpretable | Not interpretable | Serious risk | |

Risk of Bias in non-randomised studies – of interventions (ROBINS-I) tool was used for observational studies. Each domain was classified as low risk, moderate risk, serious risk, and critical risk or not interpretable. An overall bias assessment was then made using the same scale. There were no RCT’s identified for inclusion in the review.
