# Supplementary material for: Does early surgery improve outcomes for periprosthetic fractures of the hip and knee? A systematic review and meta-analysis
Source: Arch Orthop Trauma Surg. 2021 Feb 8;141(8):1393–400. doi: 10.1007/s00402-020-03739-2 (PMC8295128; doi:10.1007/s00402-020-03739-2)
Supplement: Supplementary file 10 — Supplementary file10 (DOCX 14 KB) [file 402_2020_3739_MOESM10_ESM.docx]

| **Supplementary table 2.** GRADE assessment: summary of findings | | | | | |
| --- | --- | --- | --- | --- | --- |
| Patient or population: Individuals who have sustained a periprosthetic fracture of either the hip or knee and have been managed operatively.  Setting: varied  Intervention: Early surgery (as defined by study authors)  Comparison: Delayed surgery (as defined by study authors) | | | | | |
| Outcomes | Intervention | Control | Relative effect  (95% CI) | Number of participants (Studies) | Quality of evidence |
| 30 day mortality | 11 of 1188 | 28 of 834 | RR 0.21 (0.05, 0.90) | 2022 (3) | Very low – serious imprecision and inconsistency |
| 1 year mortality | 30 of 218 | 24 of 189 | RR 0.61 (0.36, 1.03) | 407 (3) | Very low – serious risk of bias, imprecision and inconsistency |
| Length of stay | Pooled mean 3.7, Pooled SD 4.3 | Pooled mean 8.3, Pooled SD 6.6 | SMD -1.03 (-1.88, -0.19) | 1341 (2) | Very low – serious imprecision and inconsistency |
| Transfusion | 452 of 1404 | 502 of 881 | RR 0.51 (0.31, 0.82) | 2285 (4) | Very low – serious inconsistency |
| Medical complications (all cause) | 154 of 1460 | 244 of 1005 | RR 0.58 (0.42, 0.82) | 2465 (5) | Very low – serious inconsistency |
| Surgical site infection | 26 of 1460 | 34 of 1005 | RR 0.61 (0.36, 1.03) | 2022 (5) | Low – observational data |
| Reoperation (all cause) | 57 of 1188 | 71 of 834 | RR 0.63 (0.45, 0.89) | 2022 (3) | Low – observational data |

Abbreviations: GRADE = Grading of Recommendations Assessment, Development and Evaluation; CI =Confidence interval; RR =Relative risk; SD = Standard deviation; SMD = Standard mean difference
